# Bioinformatics analysis identifies ZBTB16 as a potential immune biomarker for lung cancer and pan-cancer

**DOI:** 10.3389/fgene.2025.1541732

**Published:** 2025-06-09

**Authors:** Danfei Shi, Yunxiang Cai, Di Zhu, Xinmin Li, Yong Li

**Affiliations:** ^1^ Department of Pathology, First Affiliated Hospital of Huzhou University, The First People’s Hospital of Huzhou City, Huzhou, Zhejiang, China; ^2^ Department of Clinical Laboratory, First Affiliated Hospital of Huzhou University, The First People’s Hospital of Huzhou City, Huzhou, Zhejiang, China; ^3^ Department of Clinical Laboratory, Chongqing Hospital of Traditional Chinese Medicine, ChongQing, China

**Keywords:** lung cancer, immune biomarker, DEGs, WGCNA, TCGA

## Abstract

**Objective:**

Lung cancer (LC) is a deadly cancer and a challenging public health problem worldwide. The aim of this study is to use bioinformatics to analyze the potential of ZBTB16 as an immune biomarker in lung cancer and various other cancers.

**Methods:**

Overlapping differentially expressed genes (DEGs) in LC were selected from GSE3268, GSE 1987, GSE31547 and GSE18842 gene expression data sets. We conducted a comprehensive bioinformatics analysis of these differentially expressed genes, aiming to explore their enrichment functions and pathways, relative expression levels, interaction networks, and weighted gene co expression network (WGCNA) module analysis. Then, the potential role of ZBTB16 in the occurrence and progression of lung cancer was verified, and immune invasion analysis, pan-cancer analysis and mRNA-miRNA link analysis were performed.

**Results:**

There were 16 genes with increased expression and 100 genes with decreased expression. Among them, KEGG analysis showed that these DEGs were significantly involved in complement and coagulation cascades, as well as related pathways such as proximal tubular bicarbonate recovery. Previous studies have shown that ZBTB16 plays an important role in various systemic tumors, but its function in lung cancer has not been revealed. WGCNA analysis shows that ZBTB16 is significant in lung cancer. Therefore, we will focus our attention on ZBTB16. Then, TCGA database, Human Protein Atlas database, and whole blood qPCR testing were used to verify the differential expression of ZBTB16 in lung cancer, and immune invasion analysis of ZBTB16 in lung cancer, pan cancer analysis of ZBTB16, and mRNA miRNA linkage analysis of ZBTB16 were performed.

**Conclusion:**

Three validation methods and pan cancer analysis all showed that the expression of ZBTB16 was reduced in lung cancer and various cancers, which may be a key gene for the occurrence and development of lung cancer and various cancers.

## Introduction

Global data shows that lung cancer is the leading cause of death, with China accounting for 42% of global lung cancer deaths. The prognosis of lung cancer is closely related to the initial diagnosis stage, with a 5-year survival rate of 83% for stage I patients and only 6% for stage IV patients (Luo et al., 2023; Cao et al., 2021; Houda et al., 2025). Therefore, there is an urgent need to explore effective molecular biomarkers to gain a deeper understanding of specific genes associated with lung cancer, in order to develop better diagnostic and treatment strategies.

The Gene Expression Omnibus Database (GEO), as a publicly available microarray data repository, provides convenience for bioinformatics analysis of specific gene levels in different cancer types (Wang DW. et al., 2022). The GEO database is widely used to explore differentially expressed genes (DEGs) and related molecular signals, as well as to study gene regulatory networks. However, considering the high cost and insufficient sample size of a single experiment, comprehensive analysis of different datasets and bioinformatics methods has become a necessary means to fully discover DEGs of various cancer types (Clough et al., 2024).

In this analysis, to ensure the biological relevance and translational potential of the results, we retrieved raw datasets (GSE3268, GSE 1987, GSE31547, and GSE18842) from the GEO database, all of which had sample sizes exceeding 6, ensuring the reliability of the statistical results. Meanwhile, these datasets are most suitable for the diseases currently being studied. Although they were published earlier, they still have certain reference value in terms of data quality and scientificity. In the past 5 years, relevant studies have also cited these datasets (Liu SS. et al., 2024; Teng et al., 2023; Wang F. et al., 2022).

In addition, we used the GEO2R (Clough et al., 2024) online tool for DEG screening, and subsequently constructed a protein-protein interaction (PPI) network using the selected DEGs (Lu et al., 2020). To explore possible pathogenic mechanisms, we performed Gene Ontology (GO) annotation and Kyoto Encyclopedia of Genes and Genomes (KEGG) analysis on overlapping DEGs in four datasets (Nguyen et al., 2024; Kanehisa et al., 2025). Weighted Gene Co. expression Network Analysis (WGCNA) is a systematic bioinformatics algorithm that can integrate highly correlated genes into multiple modules (Liu, 2024). This study conducted WGCNA analysis on DEGs to obtain higher reliability results.

Research has shown that ZBTB16 may play a regulatory role in immune and inflammatory responses, and studies have suggested that it may affect inflammatory responses by regulating the NF-κB signaling pathway, which has been widely studied (Zheng et al., 2021; Liu et al., 2021a; Liu et al., 2021b; Xu HJ. et al., 2024). Another study has shown that ZBTB16 is associated with various systemic tumors, and its overexpression significantly reduces malignant progression and EMT activity, which can be blocked by the expression of exogenous miR-6792–3p (Mao et al., 2024). Although ZBTB16 has been proved to have tumor suppression function in blood tumors and solid tumors (such as liver cancer and pancreatic cancer) (Chen et al., 2021), its regulatory network and immune correlation in lung cancer have not been clear. Recent studies suggest that ZBTB16 is downregulated in non-small cell lung cancer (NSCLC) and is associated with poor prognosis (Otálora-Otálora et al., 2023), but its specific mechanism still needs further exploration. Therefore, this article selects ZBTB16 as a DEGs for further research, and verifies its expression in lung cancer through clinical information from the TCGA database and quantitative polymerase chain reaction (qPCR). This in-depth exploration reveals the expression of ZBTB16 in lung cancer, paving the way for understanding the molecular mechanisms of lung cancer. Meanwhile, we also conducted extensive immune cell infiltration analysis and pan cancer analysis on ZBTB16, further exploring its potential role in various types of cancer. Through these comprehensive analyses, this study demonstrates significant innovation in identifying multiple potential biomarkers and therapeutic targets, providing new insights for future clinical applications and laying a solid foundation.

## Methods

### Data sources

The GEO dataset (Clough et al., 2024) integrated in this study (GSE3268, GSE 1987, GSE31547, GSE18842) covers different chip platforms (Affymetrix U133A, U95A, U133-Plus_2), with a total sample size of 108 tumor tissues and 79 normal controls, meeting the minimum sample size requirement (≥6 per group). Recent studies (Li Y. et al., 2024) have validated the reliability of these datasets in screening lung cancer biomarkers. Although some data were published before 2011, their consistency has been cited in subsequent omics analyses (Lei et al., 2023) to ensure the robustness of the results.

### Data processing of DEGs

Differentially expressed genes (DEGs) were extracted from the GEO database using the GEO2R method, which is an R software-based web application designed for the analysis of GEO data (Wang DW. et al., 2022). In this survey, according to |LogFC|>1 and p. adj<0.05, DEG was identified by software: R (4.2.1) and R package: ggplot2 [3.3.6]. In addition, the volcano diagram was generated using the R package: ggplot2 [3.3.6], and the Venn diagram was made using VENNY (version 2.1) to visually represent the identified DEGs (Li Y. et al., 2024).

### GO and KEGG

For a comprehensive understanding of the functional roles of the identified overlapping DEGs, we conducted Gene Ontology (GO) annotation and Kyoto Encyclopedia of Genes and Genomes (KEGG) pathway analyses using the Annotation, Visualization, and Ensemble Discovery Database (DAVID) (version 6.7) (Sherman et al., 2024; Xu S. et al., 2024; Dutta et al., 2024). GO functional annotation, a widely uszed approach, enables the grouping of genes and their protein and RNA products for comparative analysis and identification based on their biological characteristics (Nguyen et al., 2024). KEGG was employed to explore potential pathways involved in the signal transduction of these overlapping DEGs (Kanehisa et al., 2025). In our study, significance was determined based on a corrected P-value <0.05.

### Protein-protein interaction (PPI) networks of DEGs

In our investigation, we established a Protein-Protein Interaction (PPI) network (Lu et al., 2020) uszing the STRING database and visually represented it through Cytoscape (version 3.7.2).

### WGCNA

We performed WGCNA analysis (Liu, 2024) on the expression levels of these DEGs in the TCGA lung cancer dataset, using the WGCNA package in R (version 4.2.1). The data visualization was generated by the R package ggplot2 (version 3.3.6).

### Expression of hub gene proteins in the human protein atlas

The Human Protein Atlas (HPA) public database provides extensive proteomic and transcriptomic data from pathological and normal human tissue samples obtained through immunohistochemistry (IHC) and RNA sequencing analysis (Harnik et al., 2024). In our investigation, we assessed the expression levels and distribution of the ZBTB16 gene protein in lung cancer specimens and their corresponding non-cancer samples.

### Preparation of clinicopathological characteristic relationship table

In this study, we conducted univariate and multivariate Cox regression analyses using the TCGA database (https://portal.gdc.cancer.gov) to investigate the correlation between ZBTB16 and lung cancer (Li S. et al., 2024). Additionally, we generated a relationship table of clinical characteristics and a ROC curve for further exploration of the clinical significance and prognosis associated with ZBTB16 in lung cancer (Roumeliotis et al., 2024). Expression Data Acquisition: Download and collate RNAseq data from the TCGA-LUAD and TCGA-LUSC project Spliced Transcripts Alignment to a Reference (STAR) processes from the TCGA database and extract data in Transcripts Per Million (TPM) format as well as clinical data (Shen C. et al., 2024).

### Verified by qPCR experiments

In the present investigation, quantitative polymerase chain reaction (qPCR) was utilized to confirm the expression levels of the ZBTB16 gene in lung cancer. A total of 12 patients, all diagnosed with lung cancer, were recruited during their hospital admission from 1 January 2024, to 31 January 2024 (Li Y. et al., 2024). The criteria for inclusion of lung cancer patients comprised: (1) Age ≤90 years; (2) No prior history of radiotherapy or chemotherapy; (3) Absence of fever or infection within the 3 months preceding blood collection; (4) No record of blood transfusion; (5) Clinically confirmed cases of lung cancer. Meanwhile, we included 12 healthy individuals as the control group who underwent routine health examinations during the same period.

After RNA was extracted from human whole blood using a Blood Genome Purification Kit (Genefist, China), RNA was converted to cDNA using a high-capacity cDNA reverse transcription kit (Takara Bio Biotechnology (Beijing), China) according to the manufacturer’s instructions. RT-qPCR assays were performed in a 20 μL reaction mix containing 10 μL of SYBR Green Master Mix, 0.8 μL of forward primers, 0.8 μL of reverse primers, 0.4 μL of ROX Reference Dye, 2 μL of cDNA, and 6 μL of nuclease-free water. The thermal cycling conditions were as follows: 95°C for 30 s, followed by 40 cycles, 95°C for 5 s, and 60°C for 34 s.

The primer sequences used for amplifying ZBTB16 were as follows:

Forward primer; 5′-TCC​TCT​TCC​ACC​GCA​ATA​GTC​AAC-3′, reverse primer, 5′-CCG​CAT​ACA​GCA​GGT​CAT​CCA​G-3'.

The research plan has been reviewed and approved by the Medical Research and Clinical Trial Ethics Committee of the First People’s Hospital of Huzhou. All patients participating in this study provided emotional and informed consent.

### Immune infiltration

In this study, we used the “GSVA” package ssGSEA (Qi and Tang, 2024) to examine immune infiltration differences between high- and low-risk groups. Statistical analysis was performed, and significance was determined at P < 0.05.

### Pan-cancer analysis of ZBTB16

Use software R (4.2.1) to analyze the pan cancer expression of ZBTB16 in the TCGA database, and visualize it using R package: ggplot2 [3.3.6].

### miRNA analysis of ZBTB16

In this study, the miRNAs corresponding to ZBTB16 were analyzed, and the miRNAs corresponding to ZBTB16 were retrieved from three databases: TargetScan (Shen W. et al., 2024), starBase (Mathuria and Mehak, 2024), and miRwalk (Chen et al., 2024), respectively, and Venn diagram and PPI network were performed.

### Statistical analysis

Statistical analyses of the experimental data were performed utilizing R software version 4.2.1 (Govindasamy et al., 2024). The results are expressed as mean values accompanied by their standard deviations. For data that followed a normal distribution with consistent variance, the t-test was employed, whereas Wilcox’s test was applied to data exhibiting heterogeneous variance. A p-value threshold of less than 0.05 was set to determine statistical significance (McShane et al., 2024).

## Results

### Identification of DEGs

**FIGURE 1:**
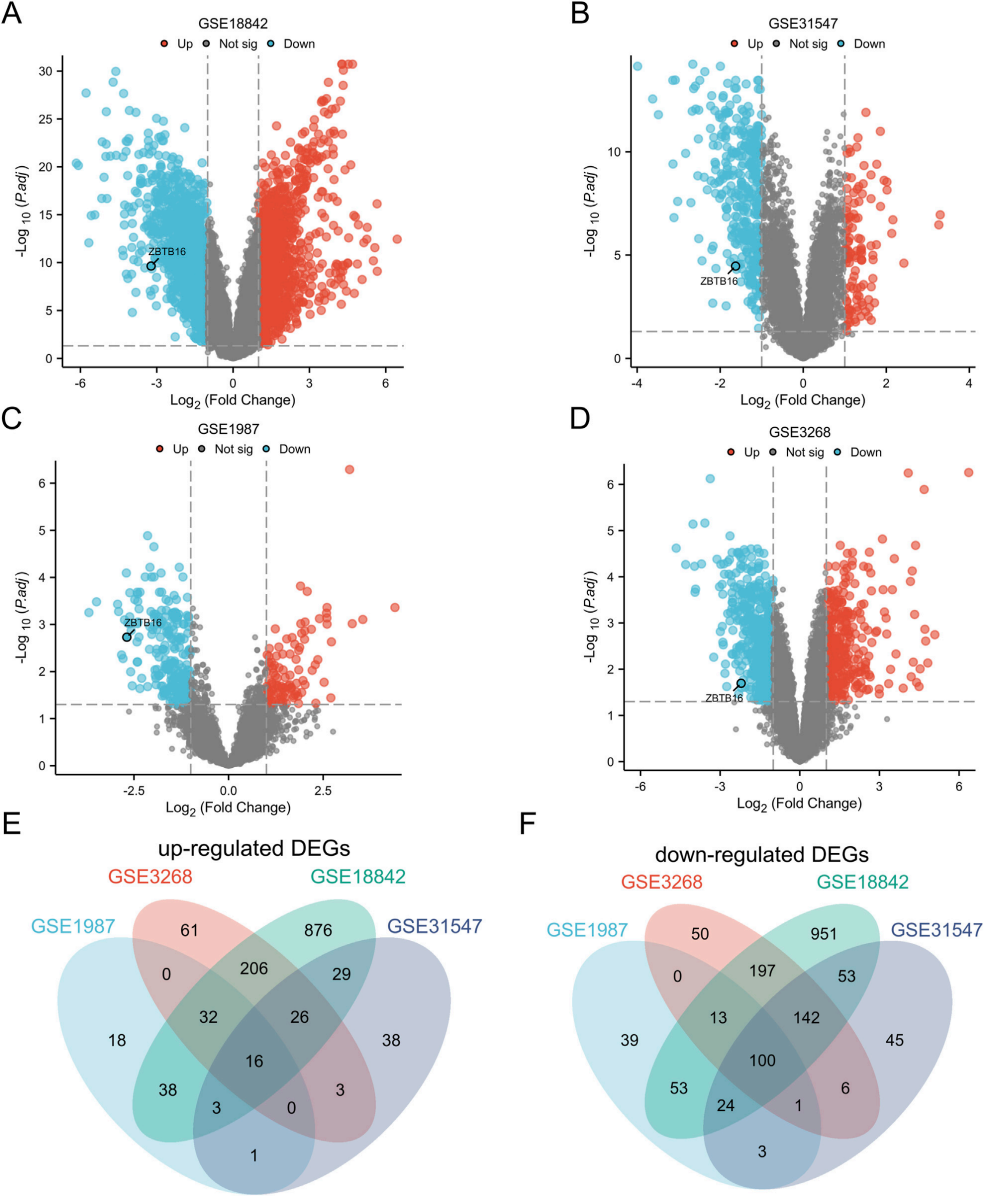
Identification of Differentially Expressed Genes (DEGs). **(A)** Map of GSE18842 volcanoes; **(B)** Diagram of GSE31547 volcano; **(C)** GSE1987 volcano map; **(D)** Diagram of GSE3268 volcano. **(E)** Venn plots of 16 overlapping upregulated DEGs in GSE3268, GSE 1987, GSE31547, and GSE18842 datasets; **(F)** Venn plots with overlapping 100 downregulated DEGs in these four datasets.

The volcano maps created for each of the four datasets in this study showed that ZBTB16 was in the downregulated portion (Figures 1A–D). Afterwards, Wayne diagram identified 116 differentially expressed genes (DEGs), of which 16 genes were upregulated and 100 genes were downregulated (Figure 1; Table 1).

**TABLE 1:** A total of 116 differentially expressed genes (DEGs).

DEGs	Gene
upregulated	*COL11A1 MMP12 SPP1 DSP SULF1 S100A2 KIAA0101 HIST1H2BD COL10A1 LAD1 ZWINT CDC20 PPAP2C THBS2 PAICS TOP2A*
downregulated	*FABP4 WIF1 CLDN5 AGER C7 FHL1 ZBTB16 MFAP4 DES CLIC5 ADIRF VWF GPX3 TNNC1 ADH1B AGTR1 TCF21 CHRDL1 ABCA8 LDB2 CDH5 FCN1 AQP1 RAMP2 PTPRB EDNRB CA4 GPM6A AQP4 LPL FCN3 ACKR1 AOC3 GPM6B DLC1 CFD CAV1 SRPX FAM107A GNG11 CD93 NR4A3 ANXA3 FXYD1 KIAA1462 EMP2 FBLN5 ADAMTSL3 FEZ1 ENG ID4 SVEP1 LDLR SMAD6 WISP2 COX7A1 SLC6A4 SPOCK2 NEDD9 ADRB2 C14orf132 TGFBR2 MYH10 TIE1 LRRC32 FOXF1 SLIT2 SPARCL1 PECAM1 DPYSL2 FBLN1 LMO2 SGCE LTBP4 ALOX5AP KAL1 FAM189A2 CAV2 THBD EFEMP1 VSIG4 MMP19 TMEM47 CALCRL RAMP3 HBEGF RASSF2 KLF4 HEG1 WFS1 PROS1 GMFG PPP1R15A CA2 FRY TACC1 MACF1 PMP22 FERMT2 ICAM2*

### GOKEGG analysis of DEGs

**FIGURE 2:**
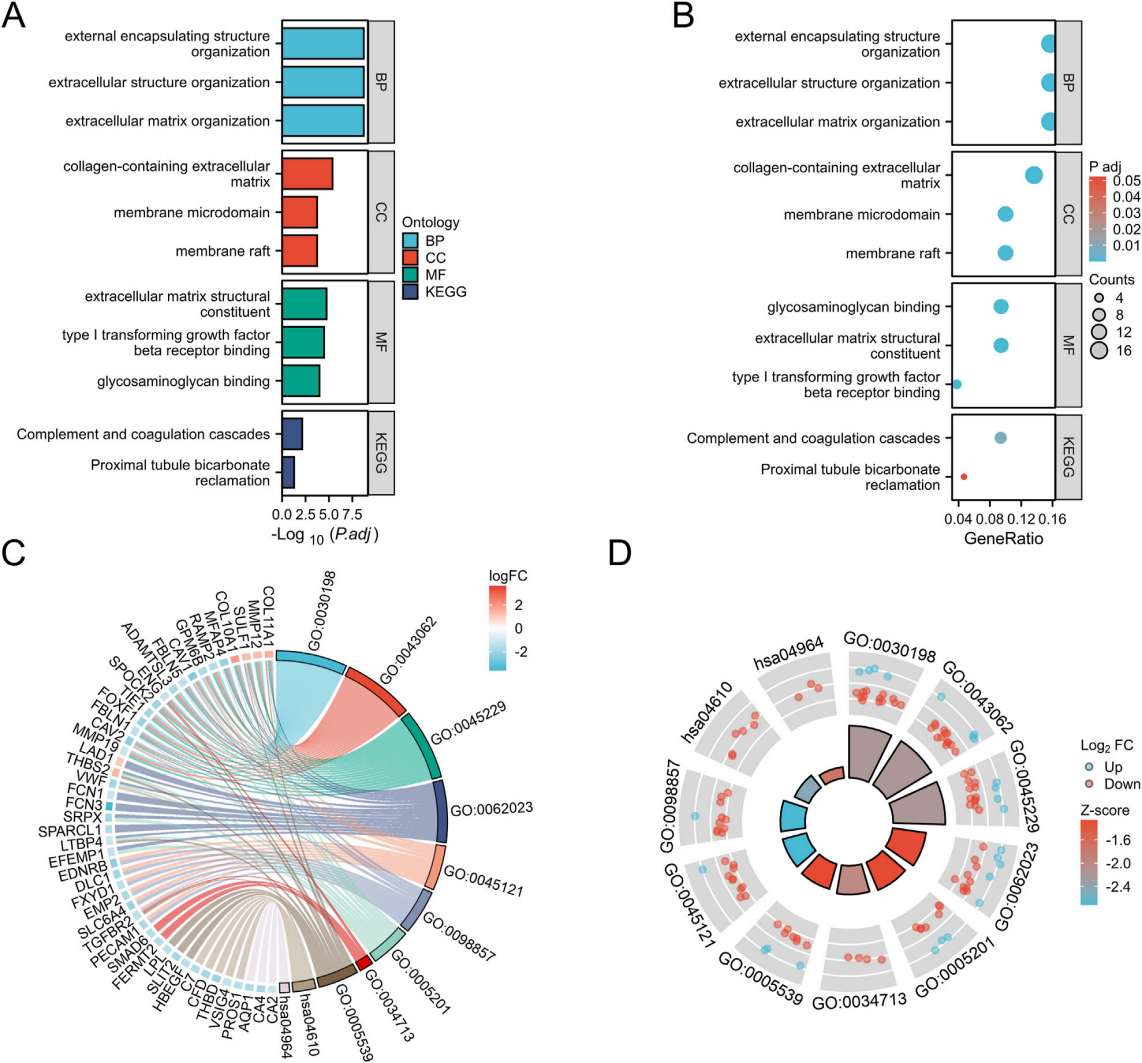
GO and KEGG analysis of overlapping DEGs in GC. **(A)**: BP, biological process; CC, cellular component; MF, molecular function; **(B)**: Kyoto Encyclopedia of Genes and Genomes (KEGG)pathway. **(C)**: GOKEGG chord diagram analysis of DEGs in dataset GSE31547. **(D)**: GOKEGG circle plot analysis of DEGs in dataset GSE31547.

To gain a deeper understanding of the overlapping differentially expressed genes (DEGs) in the four datasets, we conducted classification, functional, and pathway enrichment analysis using DAVID, with an adjusted p-value set to<0.05. In biological processes (BP), it is mainly enriched in extracellular matrix tissue, cell matrix adhesion, and angiogenesis; In the cellular component (CC), it mainly involves the extracellular matrix and membrane microdomains containing collagen; In terms of molecular function (MF), relevant terms include extracellular matrix structural components and the binding of transforming growth factor beta receptors (Figure 2A). KEGG analysis showed that DEGs were significantly involved in the complement coagulation cascade and proximal tubular reabsorption of heavy alkaloids (Figure 2B). We also combined DEGs and their LogFC values in GSE31547 data to perform GOKEGG circle plot and chord plot analysis (Figures 2C, D).

### PPI network construction

**FIGURE 3:**
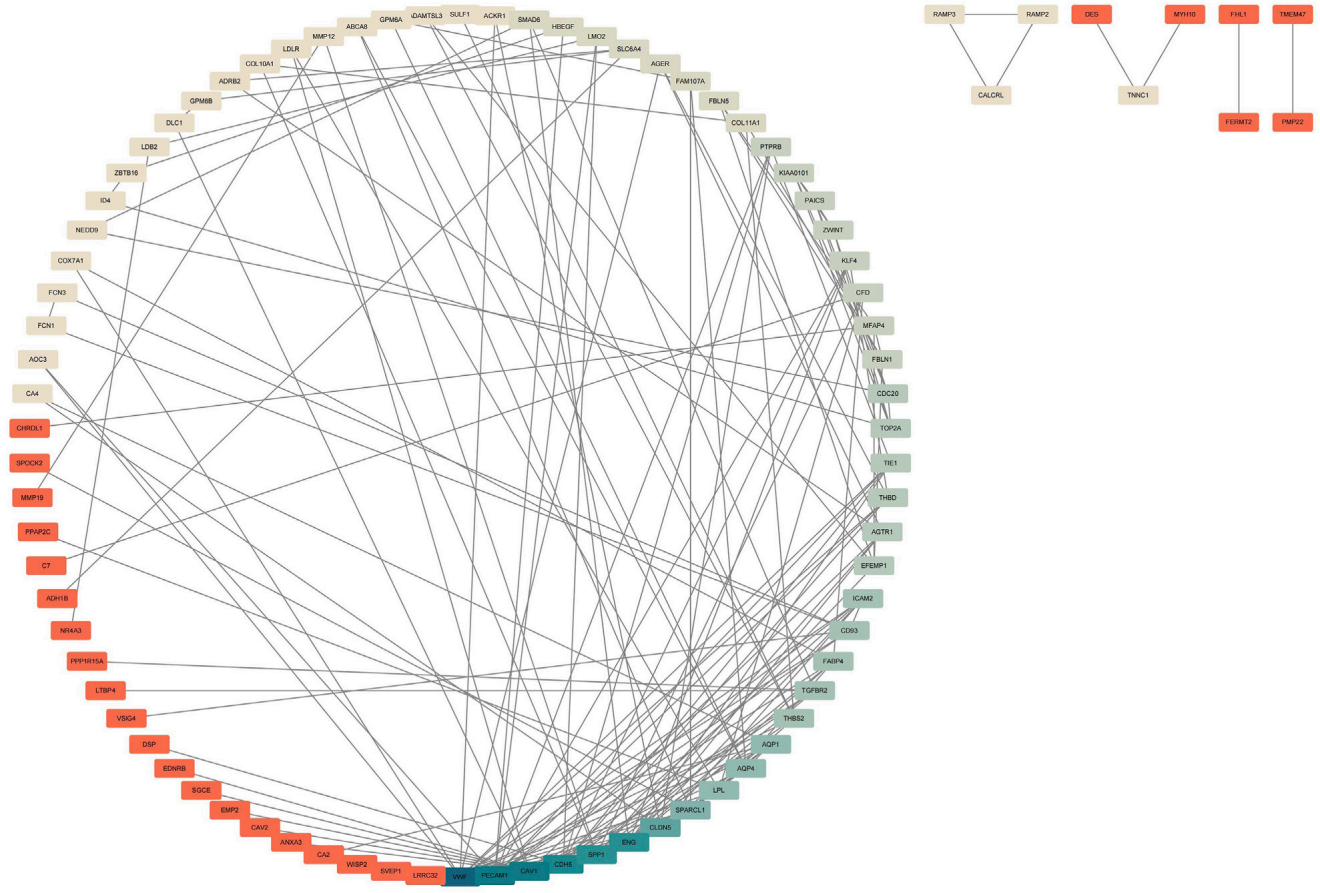
Construction of PPI Network for Common DEGs. The PPI network was established using the DEGs from the four GEO datasets. The intensity of blue color corresponds to the degree of DEGs, indicating stronger associations. The darker the blue, the higher the correlation intensity, and the darker the red, the lower the correlation intensity.

In this study, we constructed a Protein-Protein Interaction (PPI) network using the STRING database to enhance our exploration of potential relationships among Differentially Expressed Genes (DEGs). Subsequently, we employed Cytoscape for the visualization of the established PPI network (Figure 3).

### Analyzing DEGs using WGCNA

**FIGURE 4:**
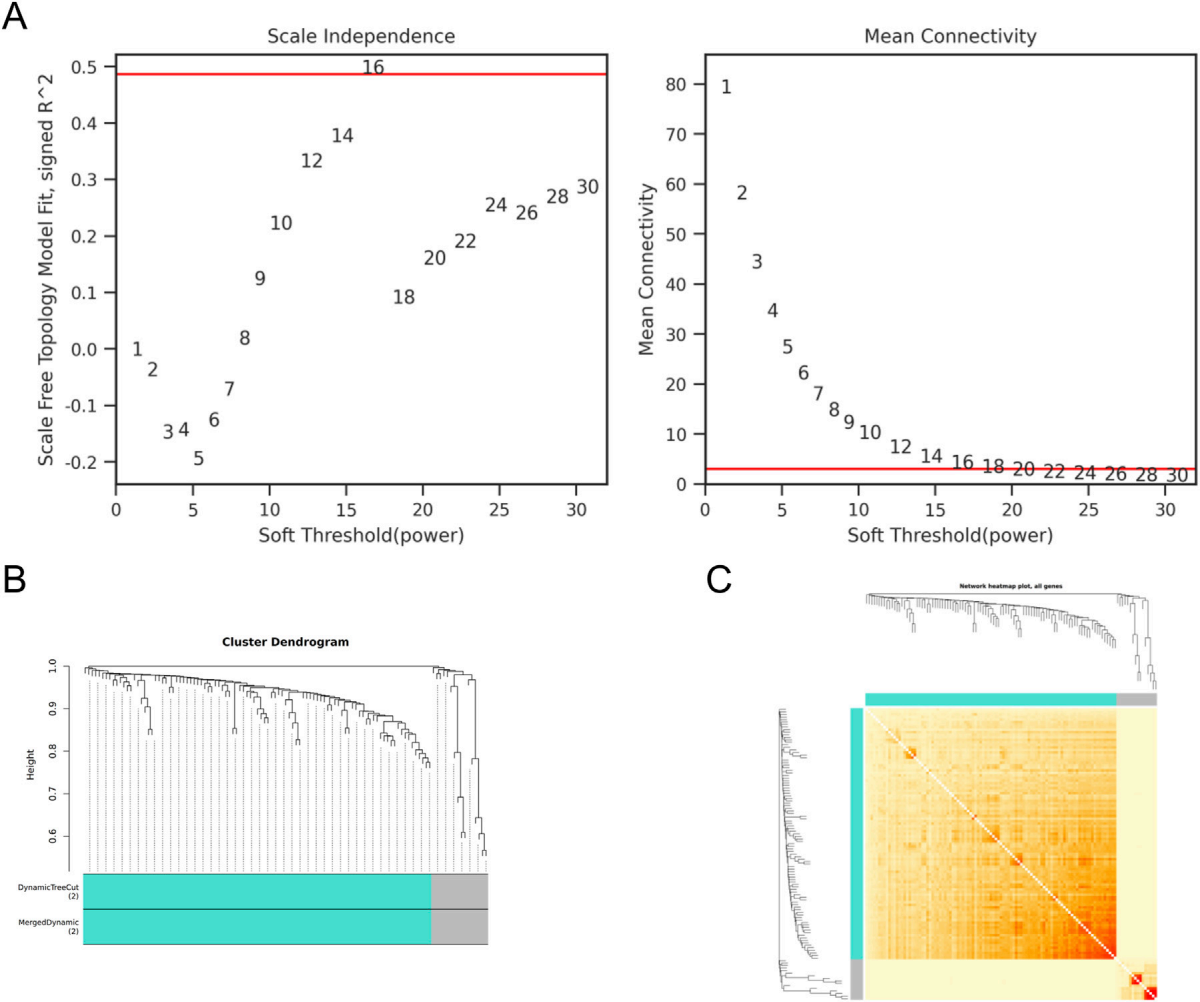
WGCNA of DEGs **(A)** Scale independence and average connectivity of DEGs. **(B)** Gene tree diagram and modules of DEGs. **(C)** Cluster heatmap of DEGs.

The gene co expression network is a scale free weighted gene network. In order to meet the distribution prerequisites of the scale free network as much as possible, it is necessary to choose the value of the adjacency matrix weight parameter power (Clough et al., 2024). The power value used in this analysis result is: 16. Based on the selected power values, WGCNA divides DEGs into two modules. The Grey module is a set of genes that cannot be classified into any module and have no reference significance, while the meaningful module contains 99 genes. ZBTB16 is at the core of this module, indicating its important role in the occurrence and development of lung cancer (Figure 4).

### Validation of TCGA and HPA databases

**FIGURE 5:**
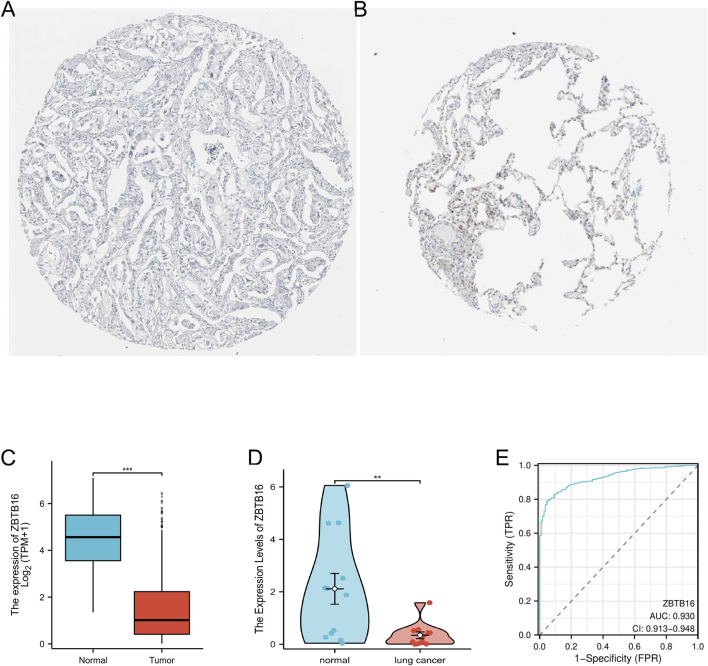
Verification of the expression level of ZBTB16 in lung cancer. **(A)** Tissue expression of ZBTB16 - Lung cancer tissue staining - Human protein profile - not detected. **(B)** Tissue expression of ZBTB16 - Staining of normal parts of the lung - Human protein profile - Medium amount. **(C)** Differential expression of ZBTB16 in lung cancer and normal control group in the TCGA database. **(D)** Expression of ZBTB16 in qPCR validation. **(E)** ROC curve of ZBTB16 in predicting prognosis in lung cancer patients.

ZBTB16 is believed to play a regulatory role in immune and inflammatory responses. We analyzed its expression in lung cancer samples, and immunohistochemistry results from the HPA database showed a decrease in ZBTB16 protein levels in lung cancer tissues, consistent with previous results (Figures 5A, B). We also conducted univariate and multivariate Cox regression analysis using the TCGA database to explore the correlation between ZBTB16 and lung cancer, including pathological T stage, N stage, age, and pathological stage. The results showed that the expression of ZBTB16 decreased with the progression of lung cancer and was statistically significant (P < 0.05, Table 2). Due to the focus of this study on the association between ZBTB16 and local and regional diffusion (T and N phases), distant metastasis (M phase) is not within the scope of this study. Analysis using R software (4.2.1) showed significant differences in ZBTB16 expression levels between lung cancer tissue and normal tissue (Figure 5C). ROC curve analysis showed that ZBTB16 is associated with lung cancer prognosis, with an AUC of 0.93, specificity and sensitivity of 95.37% and 79.06%, respectively (Figure 5E). The closer the AUC value is to 1, the better the forecasting effect. By qPCR detection of whole blood samples from lung cancer patients and normal patients in our hospital, the expression results were consistent with TCGA data and showed significant differences (P < 0.05, Figure 5D).

**TABLE 2:** Relationship between *ZBTB16* expression and clinicopathological features in lung cancer patients.

Variables	Low expression of *ZBTB16*	High expression of *ZBTB16*	P
n	520	521	
Pathologic T stage, n (%)			<0.001
T1	114 (11%)	176 (17%)	
T2	310 (29.9%)	276 (26.6%)	
T3&T4	95 (9.2%)	67 (6.5%)	
Pathologic N stage, n (%)			0.066
N0	321 (31.5%)	349 (34.2%)	
N1	129 (12.7%)	99 (9.7%)	
N2&N3	64 (6.3%)	57 (5.6%)	
Age, n (%)			0.398
≤65	234 (23.1%)	214 (21.1%)	
>65	280 (27.6%)	285 (28.1%)	
Pathologic stage, n (%)			0.003
Stage I	244 (23.7%)	297 (28.9%)	
Stage II	163 (15.8%)	124 (12.1%)	
Stage III&Stage IV	107 (10.4%)	94 (9.1%)	

### ssGSEA immunoinfiltration assay

**FIGURE 6:**
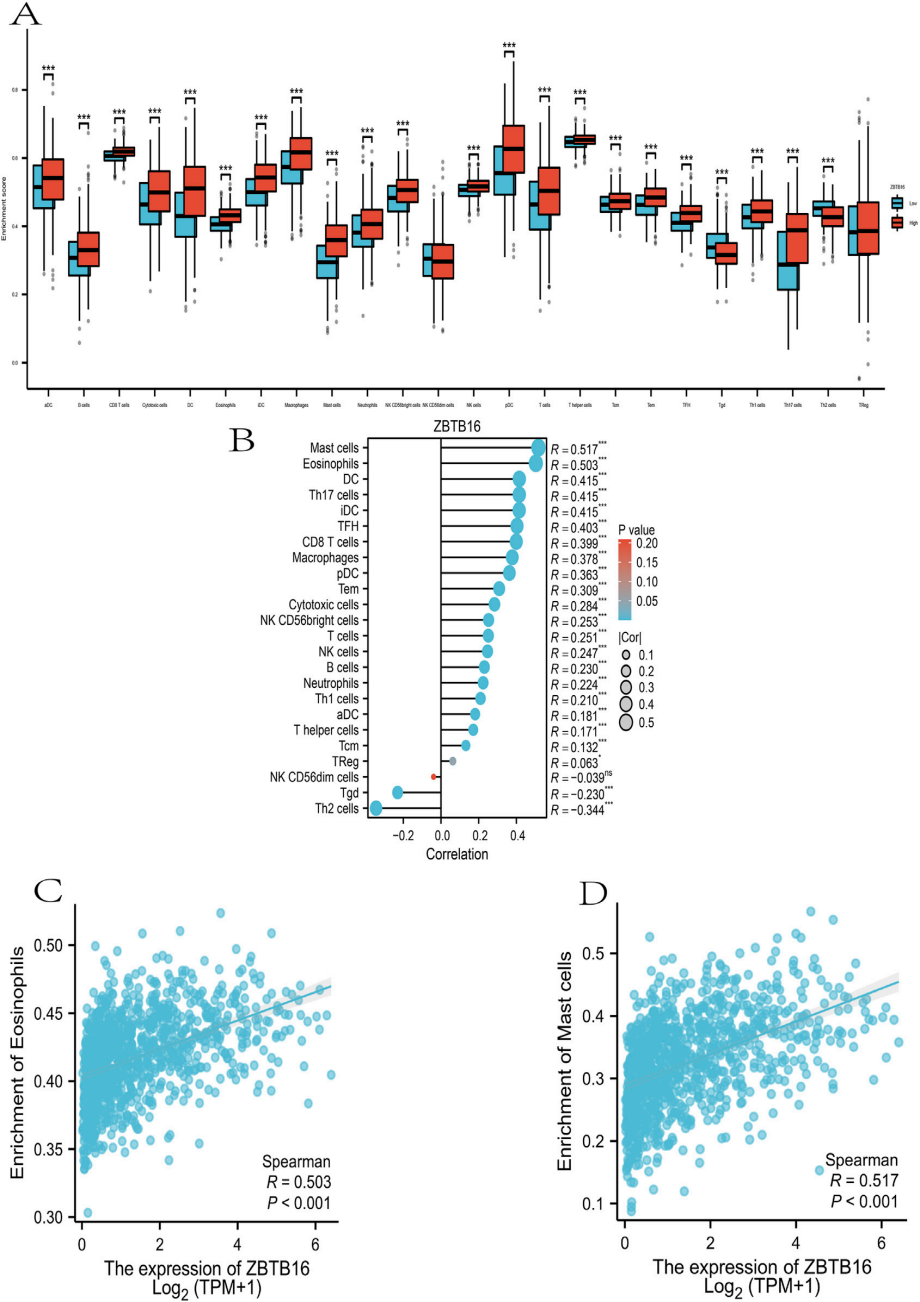
Immunoinvasive analysis of ZBTB16 in lung cancer at ssGSEA. **(A)**: ZBTB16 immunoassay was compared in all groups. **(B)**: Lollipop plot of ZBTB16 immune infiltrate-associated sex. **(C)**: Scatter plot of correlation between ZBTB16 and Mast cells. **(D)**: Scatter plot of correlation between ZBTB16 and Eosinophils.

Given the premise that prognostic variances between high- and low-risk groups predominantly stem from the impact of the body’s immune response and immune regulation, we conducted ssGSEA immunoinfiltration analysis on these groups (Nopora and Weidle, 2024). The distribution and distinctions of these cells in the high- and low-risk groups were assessed using a downloaded gene set of 28 immune cells as the reference. Refer to Figure 6, where mast cells and eosinophils exhibit a more pronounced expression difference (R = 0.517, R = 0.503). This outcome could be associated with the impacts of mast cells and eosinophils on tumor-associated cytokines and the tumor microenvironment, respectively (Sibille et al., 2022; Ghaffari and Rezaei, 2023; Ben et al., 2023). This further substantiates the immunosuppressive status associated with the high-risk group in the enrichment assay (Figure 6).

### Pan-cancer analysis of ZBTB16

**FIGURE 7:**
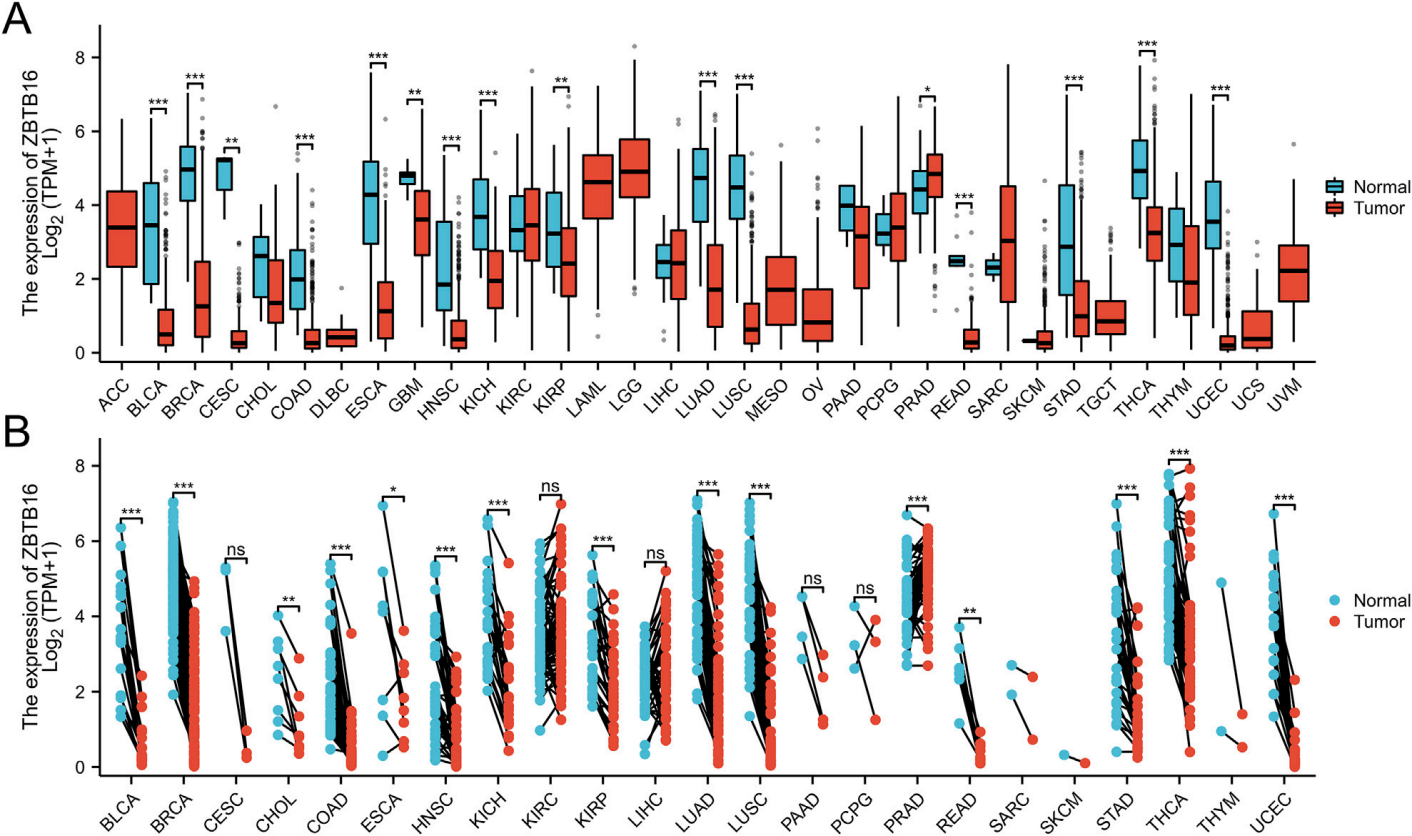
Pan-cancer analysis of ZBTB16. **(A)**: a grouped comparison of pan-cancer of ZBTB16. **(B)**: a comparison of paired samples of pan-cancer of ZBTB16.

In this comprehensive pan cancer analysis, we found the expression patterns of ZBTB16 in multiple types of cancer. The results showed that ZBTB16 was significantly downregulated in 15 types of cancer and overexpressed in one type of cancer. These observations suggest that ZBTB16 may play a role as a tumor suppressor gene in various cancers, and further research is warranted (Figure 7).

### miRNA analysis of ZBTB16

**FIGURE 8:**
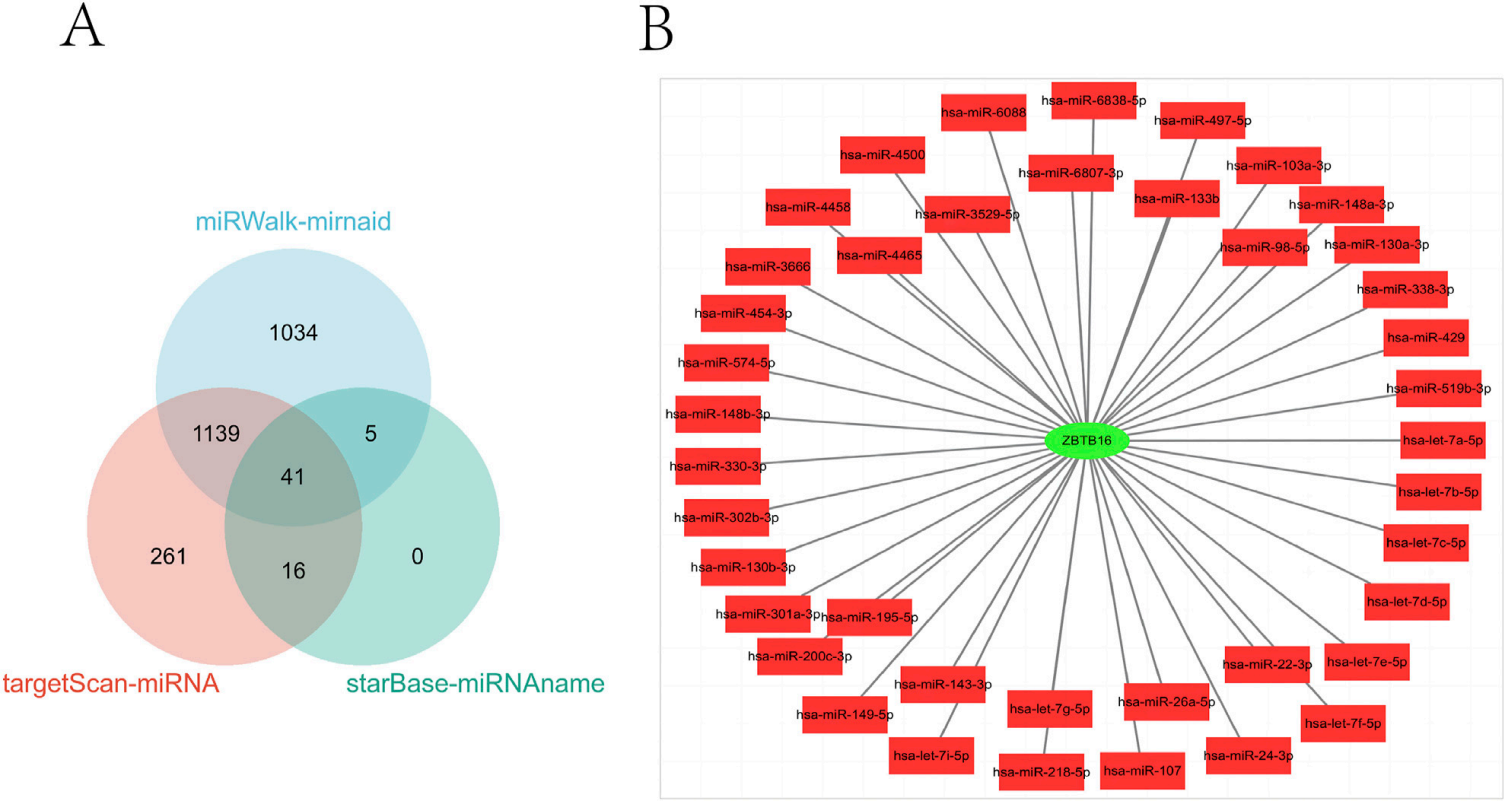
miRNA analysis of ZBTB16. **(A)**: The three miRNA databases correspond to the miRNA Venn diagram of ZBTB16. **(B)**: ZBTB16-mIRNA-PPI network diagram.

In this study, we conducted a comprehensive analysis of the microRNA (miRNA) regulatory network associated with ZBTB16. Figure 8A clearly illustrates the 41 overlapping miRNAs identified from three databases. In addition, in Figure 8B, we constructed a PPI network of ZBTB16 and miRNA using Cytoscape, which helped us understand the multi-level regulatory mechanism of ZBTB16 expression and its potential impact on cellular processes. This analysis not only emphasizes the complex interaction between miRNAs and ZBTB16, but also paves the way for further exploring the role of these miRNAs in regulating gene expression in various physiological and pathological environments.

## Discussion

Lung cancer has a high incidence rate and mortality, and is the main cause of cancer related deaths worldwide (Cao et al., 2021). About two-thirds of lung cancer cases are diagnosed in the middle to late stages, which poses a challenge to effective treatment and poor clinical prognosis (Du et al., 2022). This study analyzed four gene expression datasets from the GEO database and identified 116 differentially expressed genes (DEGs) in tumors compared to non tumor tissues, including 16 upregulated and 100 downregulated genes. Gene Ontology (GO) and Kyoto Encyclopedia of Genes and Genomes (KEGG) analysis showed that these genes are involved in extracellular matrix tissue and cell adhesion, and are associated with adhesion and ECM receptor interactions. The constructed protein-protein interaction (PPI) network revealed key DEGs. The weighted gene co expression network analysis (WGCNA) conducted through the TCGA database divided DEGs into two modules, one of which contains 99 genes, and ZBTB16 is one of them.

ZBTB16, Also known as myeloid leukemia zinc finger protein (PLZF), it was first discovered in 1993 in the study of acute myeloid leukemia, and as a transcription inhibitory factor, it may affect the tumor immune microenvironment by regulating the NF - κ B pathway (Liu D. et al., 2024). Previous studies have shown that ZBTB16 can inhibit the activation of NF - κ B (Xu HJ. et al., 2024), thereby reducing the release of pro-inflammatory cytokines and limiting tumor associated inflammatory responses. In lung cancer, low expression of ZBTB16 may lead to excessive activation of NF - κ B signaling, promoting the infiltration of immune suppressive cells (such as regulatory T cells) and forming a pro tumor microenvironment (Hu et al., 2022). In addition, ZBTB16 limits tumor metastasis in other cancers by inhibiting the EMT process (Mao et al., 2024), suggesting that it may inhibit invasive phenotypes through a similar mechanism in lung cancer. Our research found that the expression of ZBTB16 is significantly reduced in lung cancer, which is associated with disease progression. Through immunohistochemistry (IHC) staining, measurement of ZBTB16 expression levels in the TCGA database, and quantitative polymerase chain reaction (qPCR) confirmation, it was found that ZBTB16 is downregulated in lung cancer samples, indicating its potential role as a diagnostic biomarker. ROC analysis showed that the area under the curve (AUC) of ZBTB16 was 0.93, with a specificity of 95.37% and a sensitivity of 79.06%, which was superior to the research results of Guo Aixia et al. (Guo et al., 2023).

The ssGSEA immune infiltration analysis in this study showed that tumors may produce immunosuppressive cell subtypes and recruit myeloid cells that promote tumor progression, establishing a microenvironment that supports tumor progression. The key to malignant progression of tumors lies in evading immune destruction and initiating tumor cell metastasis (Han et al., 2024). We found significant differences in the expression of eosinophils and mast cells (R = 0.517, R = 0.503), suggesting that eosinophils may affect the microenvironment during tumor progression (Sibille et al., 2022; Ghaffari and Rezaei, 2023). Some studies have also found that extracellular vesicles derived from lung cancer can significantly alter the cytokines released by mast cells, which play a key role in cancer-related pathological processes (Ben et al., 2023).

This study conducted a pan cancer analysis of ZBTB16 in the TCGA database for the first time, showing that it was lowly expressed in 15 types of cancer and highly expressed in one type of cancer. In addition, for the first time, we retrieved miRNAs related to ZBTB16 from Target Scan (Shen W. et al., 2024), starBase (Mathuria and Mehak, 2024), and miRwalk (Chen et al., 2024), constructed Venn maps, and visualized the ZBTB16 miRNA-PPI network using Cytoscape. This analysis highlights the complex interaction between miRNA and ZBTB16, laying the foundation for further research on the role of these miRNAs in regulating gene expression.

This research is subject to several significant limitations that warrant consideration in future investigations. Firstly, this study aims to evaluate the diagnostic significance of ZBTB16 in lung cancer and other cancers, but only analyzed its ROC in lung cancer and did not extend to other cancers. Secondly, the validation of ZBTB16 expression in lung cancer was conducted solely through quantitative Polymerase Chain Reaction (qPCR), omitting other techniques such as Western blotting (WB) or Immunohistochemistry (IHC) for a more comprehensive validation. Thirdly, the datasets selected for this analysis lack adequate differentiation and annotation among the diverse subtypes of lung cancer, which hindered our ability to distinguish and analyze these subcategories effectively. Finally, this study used traditional methods to evaluate data before 2011, which may limit the validity of the results in relation to recent developments.

In the future, we must continue researching the following directions.

1) It is essential to conduct more extensive studies to corroborate the expression profiles of this gene across various patient demographics and cancer types, alongside their association with clinical characteristics. Furthermore, it is important to establish their potential utility as diagnostic and prognostic indicators.
2) It is imperative to broaden the scope of validation experiments and assess their expression in both animal and cellular models;
3) Multiple lung cancer subtype datasets were utilized for individual analysis to comprehend the gene’s expression in each subtype.
4) Comprehensive analysis based on a variety of latest data sets to discover more relevant key genes and propose more extensive treatment strategies.
5) Future research will include a multicenter cohort (200 lung cancer tissues and 100 normal controls) to validate the diagnostic efficacy of ZBTB16, and analyze its function in epithelial mesenchymal transition (EMT) and immune response using CRISPR/Cas9 knockout models (A549 and H1299 cell lines). In addition, the expression heterogeneity of ZBTB16 will be analyzed based on TCGA lung cancer subtype data to develop subtype specific treatment strategies.

Despite limitations, bioinformatics analysis has revealed new mechanisms and key genes that may contribute to lung cancer. Further research is needed to elucidate the regulatory roles of these genes and their potential as clinical biomarkers and therapeutic targets, providing insights for precise diagnosis and targeted therapy of lung cancer.

## Conclusion

In summary, our study revealed for the first time that ZBTB16 is a potential tumor suppressor gene in lung cancer and many other cancers, affecting cell migration, invasion, proliferation, and apoptosis. However, our study also revealed that the expression of ZBTB16 varies depending on different types of cancer, and it has the potential to be used as a biomarker for malignancy detection. Further *in vivo* studies are essential to validate the role of ZBTB16 in the development of lung cancer and other types of cancer, and understanding its molecular mechanisms is critical for the development of targeted therapies. Overall, we found a significant reduction in ZBTB16 levels in lung cancer, providing key insights for an accurate diagnosis.

## Data Availability

The original contributions presented in the study are included in the article/supplementary material, further inquiries can be directed to the corresponding author.
